# Factors Associated With Teenage Pregnancy in Tanzania: Analysis of the 2022 Tanzania Demographic and Health Survey and Malaria Indicator Survey

**DOI:** 10.3389/ijph.2025.1608146

**Published:** 2025-05-29

**Authors:** Tumaini Nyamhanga, Pankras Luoga

**Affiliations:** Department of Development Studies, School of Public Health and Social Sciences, Muhimbili University of Health and Allied Sciences, Dar es Salaam, Tanzania

**Keywords:** Tanzania, teenage pregnancy, adolescent girls, TDHS, age 15–19 years

## Abstract

**Objective:**

Few studies on teenage pregnancy in Tanzania have used a nationally representative sample. This study sought to determine the prevalence and factors associated with teenage pregnancy in Tanzania.

**Methods:**

We conducted a secondary data analysis of 3,083 teenagers aged 15–19 years drawn from the 2022 Tanzania Demographic and Health Survey.

**Results:**

After controlling for other covariates, we found that teenagers aged 18–19 years (AOR = 4.5, 95% CI:3.3, 6.1), those who said that getting permission to access healthcare was not a big problem (AOR = 2.7, 95% CI:1.4, 5.3), and those from the Southern zone (AOR = 2.4, 95% CI: 1.5, 3.9), had higher odds of reporting ever having been pregnant. Those with secondary or higher education levels (AOR = 0.3, 95% CI: 0.19, 0.39) and those who married after age 15 (AOR = 0.4, 95% CI: 0.2, 0.8), had lower odds of reporting ever having been pregnant.

**Conclusion:**

This study highlights the significant factors associated with teenage pregnancy among teenagers aged 15–19 years in Tanzania. The education system should facilitate the majority of girls obtaining at least a secondary level of education. Teenager-friendly sexual and reproductive health services need to prioritize older teenagers, with lower education levels and those from poor families.

## Introduction

Teenage pregnancy refers to pregnancy in women aged 15–19 years [1]. Over 16 million teenage girls become pregnant worldwide each year; according to recent studies, 95% of these pregnancies occur in low- and middle-income nations [2, 3]. While international teenage birth rates have decreased from 65 births per 1,000 women in 1990 to 47 births per 1,000 women in 2015, sub-Saharan Africa continues to have higher rates of teenage pregnancy [4, 5] despite several initiatives from both government and non-government organizations. The sheer number of births among teenagers is staggering. For instance, it is estimated that there were 6,114,000 births among 15–19 year-olds in sub-Saharan Africa in 2021 [6]. Tanzania’s Demographic and Health Survey (TDHS) reports show that teenage pregnancy increased from 22.8% to 26.8% between 2010/11 and 2015/16, and slightly dropped to 22% in 2022, which is still significantly high [7–9]. The prevalence of teenage childbearing varies by region in Tanzania. The five regions with the highest rates are Songwe (45%), Ruvuma (37%), Katavi (34%), Mara (31%), and Manyara (29%) [9]. This may be because Tanzania’s marriage law still allows adolescent girls to marry at age 15 with parental consent but requires boys to be 18 years old [10].

Because of the physical and psychosocial immaturity that characterizes the teenage years, pregnancy among teenagers is considered a serious reproductive health risk. It is known to result in an increased risk of maternal mortality, low birth weight, and other severe neonatal complications [3, 5, 11]. Moreover, teenage pregnancy negatively affects the physical, mental, and psychosocial wellbeing of teenagers. Studies done elsewhere in sub-Saharan Africa identified a number of factors associated with teenage pregnancy [12], namely excessive use of alcohol, substance abuse, poor educational attainment, low self-esteem, and an inability to resist sexual temptation, curiosity, and cell phone usage. Therefore, it is important that the factors associated with this major public health challenge are clearly understood in order to inform intervention policies and programmatic measures.

Understanding country-specific determinants is important in order to appropriately inform interventions that will reduce the occurrence of teenage pregnancy. However, the majority of previous studies on the determinants of teenage pregnancy were based on small samples [13, 14]. Few studies on teenage pregnancy in Tanzania have drawn from a nationally representative sample [15], and even fewer have taken a comprehensive analytical approach to determining both risk and protective factors for teenage pregnancy. Consequently, there is limited credible research evidence to inform public health programs seeking to reduce the occurrence of teenage pregnancy. Therefore, the current study seeks to fill this gap by conducting a secondary analysis of the 2022 DHS data for Tanzania to understand the prevalence and factors associated with teenage pregnancy. To the best of our knowledge, no study has reported on factors associated with teenage pregnancy using the TDHS 2022 data.

## Methods

### Data

We analyzed data from the 2022 Tanzania Demographic and Health Survey (TDHS). The survey was conducted by the National Bureau of Statistics (NBS) and the Office of the Chief Government Statistician (OCGS), Zanzibar, funded by USAID with technical assistance from ICF International and in collaboration with other national and international stakeholders. In the 2021-22 TDHS, sample selection was conducted in two stages. In the first stage, a total of 629 clusters (Enumeration Areas (EAs)) were selected. Of these, 211 EAs were from urban areas and 418 EAs were from rural areas. The second stage involved the selection of 26 households from each cluster to reach an anticipated total sample size of 16,354 households for the survey [16]. A detailed description of the survey methods, data collection process, and questionnaire is provided in the final report of the 2022 Tanzania Demographic and Health Survey (TDHS) [9].

A total of 15,699 eligible women aged 15–49 were selected, but only 15,254 women completed the interview. Our sample was further limited to girls aged 15–19, the age recognized as teenage worldwide [1, 17]. Therefore, our final analytical weighted sample included 3,083 teenage girls.

### Variables

#### Dependent Variable

The outcome variable was a binary variable indicating whether or not the individual had ever experienced a teenage pregnancy. Teenagers (15–19 years) who were currently pregnant, had a live birth, or had a pregnancy that ended in an outcome other than a live birth such as a stillbirth, miscarriage, or induced abortion were all included.

#### Main Independent Variables

Literature from sub-Saharan African countries suggests that socio-demographic and economic variables that influence teenage pregnancy include the teenager’s age, education level, marital status, type of residence, age at first sexual intercourse, parents’ marital status, the person with whom the teenager lives, and wealth quintile. [15, 18–25]^.^ Based on a literature review, nine independent variables were included in the final analysis. It is worth noting that all of these variables were measured at the time of the survey, except for age at marriage/cohabitation.

### Statistical Analysis

Due to the over- and under-sampling of households by clusters in the design of the TDHS, it was imperative to use weights to adjust for these as recommended by the DHS program. We performed all the analyses using STATA version 18, and the svyset command within STATA, which accounts for complex survey designs to ensure valid inferences. The findings were summarized using descriptive statistics, including frequencies and percentages. The association between the dependent variable [ever been pregnant] and the independent variables was established through cross tabulation and Pearson’s Chi-Square test. For the multivariable analyses, we used logistic regression to identify independent factors associated with the dependent variable, teenage pregnancy.

All the independent variables were included in the final regression model after checking for multicollinearity. This was done to determine if the independent variables were correlated with each other. Marital status was found to be significantly correlated with teenage pregnancy. This variable was modified by combining two variables, current marital status and age at marriage/cohabitation (recorded with 3 options: before 15 years, after 15 years, and never married. Multivariable regression analysis identified factors associated with teenage pregnancy while controlling for other covariates. A p-value of less than 0.05 was considered to be statistically significant.

The data used in the analysis are freely available to the public to use and no ethical approval was needed as they were obtained from the Tanzania National Bureau of Statistics by the DHS. However, permission to access the DHS datasets was sought from and granted by the Data Archivist of the Demographic and Health Surveys (DHS) Program, at https://dhsprogram.com/data/available-datasets.cfm.

## Results

### Socio-Demographic Characteristics of the Teenagers

A total of 3,083 teenage girls were involved in the study analysis, and their sociodemographic characteristics are shown in Table 1. The majority (59.8%) were aged 15–17, with a mean age of 17 years. More than half (51.9%) had a secondary or higher education level, the majority (65.4%) came from rural settings, and 40.2% of the participants came from the lake zone. Only a minority (3.3%) were married before the age of 15. Slightly less than a quarter (24.7%) of the girls came from the richest households, and more than a third (30%) came from female-headed households. Almost a third (28.8%) reported that the distance to a health facility was a big problem, while only 8.7% indicated that obtaining permission to access healthcare was a big problem.

**TABLE 1:** Sociodemographic characteristics of teenage girls aged 15–19 (Tanzania, 2025).

Sociodemographic characteristics of teenage girls aged 15–19 (N = 3,083 weighted) using TDHS 2022		
Characteristic	Frequency (N = 3,083)	Percent (%)
Age group (years)		
15–17	1,838.00	59.6
18–19	1,245.00	40.4
Highest education level		
Primary or less	1,484	48.1
Secondary or higher	1,599	51.9
Type of place of residence		
Urban	1,068	34.6
Rural	2,015	65.4
Geographical zones		
Lake zone	1,245	40.4
Northern zone	341	11.1
Central zone	355	11.5
Southern zone	540	17.5
Coastal zones	603	19.6
Marital status		
Not married	2,519	81.7
Married	564	18.3
Respondent currently working		
Not working	2,164	70.2
Yes, working	920	29.8
Wealth quintile		
Poorest	524	17
Poorer	538	17.4
Middle	635	20.6
Richer	625	20.3
Richest	761	24.7
Distance to a health facility		
Big problem	887	28.8
Not a big problem	2,196	71.2
Getting permission to go to a healthcare facility		
Big problem	268	8.7
Not a big problem	2,815	91.3
Ever having been pregnant		
No	2,405	78
Yes	678	22

### Factors From the Bivariate Model Associated With Teenage Pregnancy

Of all the teenage girls (15–19 years) included in our survey, almost a quarter (22%) reported having been pregnant, as shown in Table 2. Of all the teenagers who reported ever having been pregnant, 39.5% were aged 18–19 years old and 10.1% were aged 15–17 years old. Age was significantly associated with teenage pregnancy. A higher percentage of teenage girls with a primary education or less had a pregnancy compared to those with a secondary education or higher (35.6% vs. 9.3%). Place of residence was also significantly associated with ever having a teenage pregnancy, as 24.9% of the girls from rural areas had a teenage pregnancy compared to 16.4% of those from urban areas who reported ever having been pregnant. Geographical zones were significantly associated with teenage pregnancy, with the highest (29.1%) in the Southern zone, followed by 23.1% in the Lake zone, the Central zone (22.2%), and the coastal zones (18.3%). The lowest percentage (12.8%) was in the Northern zone. We found a significant association between age at marriage/cohabitation and ever having been pregnant: the majority (88.2%) of those who married/cohabited before age 15 reported ever having been pregnant, while 77.5% of those who married/cohabited after age 15 reported ever having been pregnant. Only 7.5% of those who never married reported ever having been pregnant. The percentage of those who had ever had a teenage pregnancy decreased with increased wealth, ranging from 33.9% among those from the poorest households to 13.1% among those from the richest households; the wealth quintile was significantly associated with the outcome variable. Among those respondents who were from female-headed households, 20.1% had a teenage pregnancy, while 22.8% of those from male-headed households reported ever having been pregnant. However, the sex of the household head was not significantly associated with teenage pregnancy. Among those who responded that distance to a health facility was a big problem, 24.2% had a teenage pregnancy, while 21.1% of those who responded that distance to a health facility was not a big problem had a teenage pregnancy. Distance to a health facility did not show any significant association with teenage pregnancy. Similarly, 17.6% of the respondents who said that getting permission to access healthcare was a big problem had experienced teen pregnancy compared to 22.4% of those who said that getting permission to access healthcare was not a big problem. However, no significant association was found with the outcome variable. Age, education level, geographical zone, age at marriage/cohabitation, type of place of residence and wealth quintile were found to be significantly associated with having ever been pregnant.

**TABLE 2:** Bivariate analysis using Chi-square to determine the association between sociodemographic factors and teenage pregnancy (Tanzania, 2025).

Bivariate analysis using TDHS 2022			
Ever had a teenage pregnancy	Yes		
	Percent	95% CI	p-value
Age groups			
15–17	10.1	[8.5,11.9]	0.001
18–19	39.5	[35.8,43.4]	
Highest education level			
Primary or less	35.6	[32.5,38.9]	0.001
Secondary or higher	9.3	[7.7,11.2]	
Type of place of residence			
Urban	16.4	[13.1,20.4]	0.001
Rural	24.9	[22.3,27.7]	
Geographic zones			
Lake zones	23.1	[19.7,27.0]	0.002
northern zone	12.8	[8.7,18.5]	
Central zone	22.2	[15.6,30.6]	
Southern zone	29.1	[25.0,33.5]	
Coastal zone	18.3	[14.2,23.3]	
Married status			
Not married	9.1	[7.6,10.7]	0.001
Married	79.6	[75.4,83.3]	
Respondent currently working			
No	17.8	[15.4,20.4]	0.001
Yes	31.8	[28.2,35.7]	
Wealth index			
Poorest	33.9	[28.8,39.5]	0.001
Poorer	26.5	[22.3,31.1]	
Middle	24.1	[20.3,28.2]	
Richer	16.7	[13.4,20.7]	
Richest	13.1	[9.3,18.3]	
Distance to a health facility			
Big problem	24.2	[20.5,28.2]	0.179
Not a big problem	21.1	[18.7,23.8]	
Getting permission to go to a healthcare facility			
Big problem	17.6	[12.9,23.6]	0.127
Not a big problem	22.4	[20.2,24.8]	

### Factors Associated With Teenage Pregnancy Among Adolescent Girls Aged 15–19

All factors from the bivariate model were fitted in the multivariate logistic regression model to determine the strength of the association and control for possible confounders. After controlling for other covariates, as shown in Table 3, teenage girls aged 18–19 years had 4.5 times (AOR; 4.5, 95% CI:3.3, 6.1) the odds of reporting ever having been pregnant compared to those aged 15–17. Those with a secondary education or higher had 0.3 times (AOR; 0.3, 95% CI: 0.2, 0.4) lower odds of reporting having ever been pregnant compared to those with primary education or less. Those who married after age 15 had 0.4 times (AOR; 0.4, 95% CI:0.2, 0.8), while those who were never married had 0.01 times (AOR; 0.01, 95% CI: 0.01, 0.02), lower odds of reporting having ever had a teen pregnancy compared to those married before the age of 15. Teenagers living in rural settings had similar odds of ever having a pregnancy compared to those living in urban areas. Those from the Southern zone had 2.4 times (AOR; 2.4, 95% CI:1.5, 3.9) higher odds of reporting ever having had a pregnancy compared to those from the Lake zone. Only the southern zone was significant among all zones. Those from the richest households had 0.7 times (AOR; 0.7; 95% CI: 0.4, 1.2) lower odds of reporting ever having been pregnant compared to those from the poorest households. Those from female-headed households had 1.6 times (AOR; 1.6; 95% CI: 1.2, 2.3) higher odds of reporting ever having a pregnancy compared to those from male-headed households. Those who responded that distance to a healthcare facility was a big problem had 1.2 times (AOR; 1.2; 95% CI: 0.8, 1.7) higher odds of reporting ever having a pregnancy compared to those who responded that distance to a healthcare facility was not a big problem. Those respondents who said that getting permission to access healthcare was a big problem had 2.7 times (AOR; 2.7; 95% CI:1.4,5.3) higher odds of reporting having ever had a teen pregnancy compared to those who said that getting permission to access healthcare was not a big problem.

**TABLE 3:** Adjusted odds ratios of teenage pregnancy among girls aged 15–19 (Tanzania, 2025).

Multivariable logistic regression showing the factors associated with teenage pregnancy among adolescent girls aged 15–19 in Tanzania using the 2022 TDHS			
Sociodemographic factors	aOR	95% CI	P-value
Age group (Years) (Ref:15–17 age group)			
18–19	4.52	3.36, 6.06	0.001
Highest educational level (Ref: primary or less)			
Secondary or higher	0.27	0.19, 0.39	0.001
Type of place of residence (Ref: Urban)			
Rural	1.03	0.71, 1.49	0.877
Marital Status (Ref: Not married)			
Married	25.29	17.73, 36.08	0.001
Geographical zones (Ref: Lake zone)			
Northern zone	0.53	0.28, 1.03	0.06
Central zone	1.17	0.66, 2.1	0.585
Southern zone	2.07	1.36, 3.15	0.001
Coastal Zones	1.41	0 .85, 2.34	0.179
Respondent currently working(Ref: Not working)			
Yes	1.19	0.85, 1.66	0.304
Wealth index(Ref: Poorest)			
Poorer	0.9	0.55, 1.48	0.669
Middle	0.75	0.49, 1.15	0.187
Richer	0.6	0.37, 0.95	0.031
Richest	0.57	0.34, 0.95	0.031
Distance to a healthcare facility (Ref: Big Problem)			
Not a big problem	1.2	0.83, 1.75	0.336
Getting permission to go to a healthcare facility (Ref. Big problem)			
Not a big problem	2.66	1.41, 5	0.003

## Discussion

The study aimed to determine the factors associated with teenage pregnancy among teenage girls aged 15–19 years in Tanzania using the 2022 TDHS. The study found that the factors associated with reporting ever having been pregnant included: belonging to the 18–19 age group, having been married before the age of 15, having never been married, having a primary education level or lower (primary school or less), and belonging to the poorest and poor households. Although the 22% prevalence of teenage pregnancy reported in the 2022 DHS for Tanzania showed a decline from the 27% reported in the country’s 2015/2016 DHS, it is still unacceptable. This suggests that Tanzania needs to further intensify its efforts to reduce the incidence of teenage pregnancy.

Regarding the factors analyzed in this study, the finding that older teenagers were more likely to become pregnant compared to younger teenagers is in agreement with other studies conducted in Africa [26–28]. This could be attributed to the fact that teenagers aged 18–19 are subject to both internal (physiological) and external (socio-environmental) pressures to have sex after being exposed for a much longer period post menarche compared to younger teenagers [27].

Regarding education, this study has shown that teenagers with a secondary education or higher had 70% lower odds of ever having had a pregnancy compared to those with a primary or no formal education, which is in agreement with findings reported in similar studies in Tanzania and other East African countries [15, 21]. This may be because the higher the educational achievement, the higher the level of empowerment that enables women to have control over their reproductive destiny. On the other hand, pregnant teenagers may not continue their formal education, which negatively affects their educational attainment. This may be due to strict laws that prevent pregnant girls from continuing their education, or it could be because pregnant or postpartum teenagers are not able to pay for their education. This may be because parents consider them grown up or move to establish their families after getting pregnant, hence thought to be independent [29]. Our study therefore suggests that programs targeting to improve the education of teenage girls could reduce the number of teenage pregnancies among this subpopulation.

Regarding place of residence, our analysis has revealed that teenagers residing in rural settings had similar odds of reporting ever having been pregnant compared to their urban counterparts. This shows that the area of residence, whether rural or urban, is not associated with teenage pregnancy. These findings are surprising to us as we expected teenagers from rural settings to have higher odds of reporting ever having been pregnant because rural areas in sub-Saharan Africa are characterized by poverty which impairs access to and utilization of contraceptive information and forces poor girls to engage in sex as a way to meet their basic needs. This contradicts previous findings from studies conducted in sub-Saharan Africa [12, 21, 30].

Teenage girls who were married after age 15 had lower odds of reporting ever having been pregnant compared to their counterparts who were married before the age of 15. In addition, we found that those who never married had almost zero (0.01) odds of reporting ever having been pregnant compared to their counterparts who were married before the age of 15. Similar findings have been reported in other studies using DHS data [15, 23]. These findings indicate that early marriage may expose teenagers to pregnancy in three ways. First, married teenagers become preoccupied with procreating to meet the expectations of their husbands and in-laws [31]. Second, their freedom to practice contraception becomes limited [32] as they often marry much older men who take control over decision-making [33]. Third, some parents force their daughters to marry due to a teenager’s premarital pregnancy to avoid societal shame. These findings may have policy implications for spearheading the efforts to combat child marriage in society as laws regarding marriage in Tanzania remain controversial. The Sexual Offences Special Provisions Act (SOSPA) number 4 of 1998 [34] was enacted to protect women and children from sexual violence. However, a critical examination of the Act shows that teenage girls are legally protected against rape so long as they are not married. When a married teenage girl is 15 or older, sexual intercourse with her “husband” is considered legal. This is because Tanzania’s marriage law allows teenage girls to marry at age 15 [10, 35]. One of the major weaknesses in using marital status as a variable is the inability to clearly account for the timing of when the marriage or pregnancy happened. By including age at marriage/cohabitation, our analysis shows that more teenage girls who got married before the age of 15 reported teen pregnancy compared to those who married after the age of 15 or those who never married.

Teenage girls from the richest households were less likely to have a teenage pregnancy than those from the poorest households. This finding is in line with the previous studies reported elsewhere in Africa [12, 21, 30]. This may be explained by the fact that poor parents are often unable to sufficiently meet their daughters’ basic needs such as food, clothing, soap, body oil, school materials, etc. [14]. Consequently, these girls may engage in sex in exchange for money or promises of money out of necessity as they struggle to meet their basic needs [36]. Similar findings were reported in Sierra Leone and other parts of sub-Saharan Africa where teenagers from the richest households were less likely to report having ever been pregnant compared to their counterparts coming from the poorest, poorer and middle-income households [4, 23].

### Strengths and Limitations of the Study

The study drew its strengths from the large sample size of the DHS, which provides findings that are nationally representative and generalizable. However, the cross-sectional nature of the study limited the ability to establish causal-effect relationships between the independent and dependent variables.

The study analyzed the secondary data; therefore, it could inherently include errors associated with the sampling design and other biases committed during data collection. Nevertheless, the use of national survey data and the application of weighting eliminated biases typically associated with sampling and measurement.

Moreover, due to the cross-sectional nature of the survey, this study assessed the association between teenage pregnancy which had occurred in the past (prior to the survey) and explanatory variables that were measured during the survey. Consequently, we were unable to establish the temporal relationship between teenage pregnancy and the explanatory variables with the exception of age at marriage/cohabitation.

### Conclusion

This study shows that the high prevalence of teenage pregnancy in Tanzania is associated with several factors; belonging to the 18–19 age group, having lower educational achievement (primary school or less), marrying before the age of 15, belonging to the poorest households, having a female head of household and reporting that it is not a big problem to get permission to access healthcare. These findings have policy and programmatic implications. First, the current legal reforms seeking to stop child marriage before the age of 15 should be strengthened. Second, the education system should facilitate the majority of girls completing at least a secondary level of education. Finally, teenager-friendly sexual and reproductive health services need to prioritize older teenagers, with a lower education level, and those from poor families.

### Recommendations for Further Studies

Conducting a qualitative study is crucial to ascertain the reasons from teenage girls themselves and to provide in-depth information on some of the aspects that could not be captured using DHS data, such as how these variables lead the teenagers to have a pregnancy.
